# Psychometric properties of the SocioEmotional Skills Instrument for Teachers using network approach: English and Spanish version

**DOI:** 10.3389/fpsyg.2024.1421164

**Published:** 2024-09-20

**Authors:** Fabiola Sáez-Delgado, Javier Mella-Norambuena, Yaranay López-Angulo

**Affiliations:** ^1^Departamento Fundamentos de la Pedagogía, Facultad de Educación, Universidad Católica de la Santísima Concepción, Concepción, Chile; ^2^Departamento de Ciencias, Universidad Técnica Federico Santa María, Concepción, Chile; ^3^Departamento de Psicología, Facultad de Ciencias Sociales, Universidad de Concepción, Concepción, Chile

**Keywords:** network approach, socioemotional skills, psychometric, teachers, validation

## Abstract

The importance of socioemotional teaching skills has been highlighted for its link with better academic, social, emotional, and behavioral results of students, as well as for its contribution to the work wellbeing, mental health, and prosperity of teachers. However, there are few instruments that measure these skills in teachers in the context of their professional practice. The purpose of this research was to analyze the psychometric properties of the socioemotional Skills Instrument for Teachers (SEMS-IT). An instrumental design and a sample of 853 Chilean secondary school teachers were used. To evaluate the dimensional structure of the instrument, a portion of the sample (*n* = 468) underwent a network estimation method with exploratory graph analysis (EGA) using a Gaussian GLASSO model. Then, in order to confirm the structural consistency and stability of the items, the analysis was replicated in a second sample (*n* = 385), where these results were additionally contrasted with those of the confirmatory factor analysis (CFA). The EGA findings confirmed a structure of four dimensions and 19 items in total: (a) cognitive management of teacher emotion (four items), (b) teacher empathic concern (four items), (c) teacher–student relationship (four items), and (d) adverse classroom climate (seven items), with a 7-point Likert scale response format. The CFA showed good and acceptable fit indicators, *X^2^*(171) = 354.546 (*p* < 0.001), Comparative Fit Index (CFI) = 0.971, Tucker–Lewis index (TLI) = 0.966, Root Mean Square Error of Approximation (RMSEA) = 0.061, and Standardized Root Mean Square Residual (SRMR) = 0.062. In conclusion, a tool for the assessment of teachers’ socioemotional skills, valid for school-based educational research, is provided. Implications of the findings at the theoretical and practical levels are discussed, as well as limitations and future projections for future research.

## 1 Introduction

### 1.1 Importance of socioemotional skills for teachers

Understanding and delimiting the concept of Social–Emotional Skills (SEMS) is not a simple task, as it is frequently used as a generic term that allows people to express, regulate and understand their thoughts, emotions, and behaviors in everyday situations and interactions with others, as well as to adapt to changing conditions (Schoon, 2021). In the present study, situated in the school context, SEMS are defined as the effective deployment of strategies that enable teachers to handle/manage complex situations, both interpersonally and intrapersonally, in a way that promotes their own well-being and that of their students, positively impacting socioemotional development—mutual/collective/group. Teachers’ SEMS have high associations with positive outcomes in social, behavioral, affective, academic, and student well-being outcomes (DeLay et al., 2016; Roorda et al., 2017). Recent studies have also shown that these skills function as a protective factor for teachers’ mental health, professional achievement, well-being, and thriving (Oliveira et al., 2021; Ornaghi et al., 2023; Sáez-Delgado et al., 2023; Sánchez-Pujalte et al., 2021; Serrano-Díaz et al., 2017; Zhang et al., 2023).

### 1.2 Models for the study of SEMS in teachers

There are several theoretical models in the socioemotional field (Fitzgerald et al., 2022; Muzzio and Strasser, 2022). Among the referent models are Mayer and Salovey’s emotional intelligence model 2000 (Mayer et al., 2000) and Bar-On (1997, 2006); both focused on the concept of emotional intelligence. Gross (1998) emphasizes the concept of emotional regulation. Bisquerra and Peréz (2007) is linked to the concept of emotional competencies. Collaborative for Academic, Social, and Emotional Learning (CASEL) (2008, 2013) includes the concept of social and emotional learning (SEL). Frameworks include that of Jones and Bouffard (2012); there is also Schonert-Reichl’s (2017) three-component framework on SEL that includes three distinct and interrelated dimensions (learning context, student SEL, and teacher SEL). Other models, such as the prosocial classroom model (Jennings and Greenberg, 2009), include five teaching consciences as a framework for understanding teachers’ SEC, where it is emphasized that with awareness comes competence (Rodriguez et al., 2020); the Social and Emotional Competence School Model of Collie (2020); and the DOMASEC-Domains and manifestations of socio-emotional competences of Schoon (2021), which focus specifically on social–emotional competences (SEC).

The delimitation and characterization of these models can be done by considering two central aspects. First, a key aspect of the models is the areas or contexts in which they have been formulated and/or mainly implemented, some of which have been applied in a variety of contexts, such as labor, clinical, educational, and organizational. Second, another key aspect of the models is the central construct that they define since the diversity and breadth of principles on which they are based are recognized, which vary between models of capacity, competence, trait and ability. However, the ability models stand out for having the potential to learn, that is, they allow socioemotional development, versus those models, such as trait models, that include factors that do not really change over time (they are stable), and, therefore, will not change with an intervention. The above background delimited the selection of specific theoretical models for the design of the instrument of this study. The models of Jennings and Greenberg (2009), Collaborative for Academic, Social, and Emotional Learning (CASEL) (2008, 2013), and Collie (2020) were mainly considered because, on the one hand, they describe integral socioemotional skills and, on the other hand, they have been focused on educational and school contexts, highlighting the importance of adapting and applying these theoretical models according to the needs and characteristics of the environment in which they are to be implemented, in this case specifically, focused on the socioemotional skills needed by teachers for the success of their professional practice.

### 1.3 Instruments used for the measurement of teachers’ SEMS

In empirical studies on teachers’ SEMS, it is possible to identify the use of different self-report instruments, among which the following stand out: Trait Meta-Mood Scale (TMMS), based on the model of Salovey and Mayer (1990), which assesses emotional intelligence in the dimensions of emotional attention, emotional clarity, and emotional repair; Petrides (2009) Trait Emotional Intelligence Questionnaire (TEIQue), an instrument that combines scales of emotion regulation and relationship management skills, which measures the degree to which subjects perceive themselves as controlling their own emotions, how they influence other people’s feelings, how they assert themselves, and how they establish positive relationships with others; Yoder’s (2014) Self-Assessing Social and Emotional Instruction and Competencies which measures teaching practices that affect students’ SEL and own SECs to implement those teaching practices with students; The Social and Emotional Competencies Questionnaire (SEC-Q) (Zych et al., 2018), which includes four scales (self-awareness, self-awareness and motivation, social awareness and prosocial behavior, and responsible decision making); and the EduSEL which is a multidimensional self-report instrument to assess educators’ SEL competencies (Hemi and Kasperski, 2023). A review of these instruments reveals significant limitations related to the age group and context for which they were originally designed. Although applied to teachers, many were originally designed for adolescents and adults in general (e.g., see Salovey et al., 1995; Bar-On, 1997; Mayer et al., 2002; Mayer et al., 2003; Zych et al., 2018), others were focused on clinical populations (e.g., see Bar-On, 1997; Gross and John, 2003; Pérez-Escoda et al., 2021). However, instruments specifically developed for teachers, addressing SEL and essential teaching skills, are limited/scarce (e.g., see Hemi and Kasperski, 2023; Aldrup et al., 2020; Yoder, 2014) and mostly focused on general SEL, but not on crucial, specific, delimited skills necessary for teaching success. Thus, it can be argued that there is a need to validate specific instruments for teachers that include variables relevant to the educational context and items that fit the classroom routine.

A relevant aspect to consider is that most psychometric studies in the area of educational psychology have used the traditional exploratory and confirmatory factor analyses, but recently, the so-called network analysis models, also known as network psychometrics, have caught the interest of researchers (Christensen et al., 2019). One of the potential differences between models that include latent variables is that they conceive of observable variables as the product of unobservable latent factors, whereas network analysis models implement graph theory to construct a network that can represent the different associations between observable variables (Cai et al., 2020). Therefore, the structural characteristics of network models exponentially enrich the possibility of revealing the multiple relationships between variables in a dynamic system, providing a new perspective for the visualization and study of various current phenomena in educational psychology. This type of analysis responds assertively to the complexity of the analyzed variables of human beings and its valuable contributions favor modelling to improve the proposal of psychometric instruments, in this case, to understand teacher SEMS (Borsboom, 2022).

### 1.4 Essential teacher’s SEMS

It is indisputable that teaching SEMS positively impacts the socioemotional development of their students within the school environment, acting as role models in social and emotional skills, norms, and behaviors. School, as the primary socialization environment after home, underscores the importance of educators promoting healthy interactions and effectively managing the learning environment (Schoon, 2021; Lechner et al., 2019; Lee et al., 2016). Although there is a wide variety of SEMS, among those that are critical for teachers, the following inter- and intrapersonal skills stand out: (a) cognitive management of teacher emotion (CMTE), (b) teacher empathic concern (TEC), (c) teacher–student relationship, and (d) Adverse Classroom Climate (ACC; Lechner et al., 2019; Martínez-Yarza et al., 2023; Scheirlinckx et al., 2023).

#### 1.4.1 Cognitive management of teacher emotion

Gross’s (1998, 2002) model of emotion regulation illustrates how people influence their emotions, determining which emotions they experience and when and how they express them. The importance of cognitive management of emotions is highlighted here because it addresses one of the most reported aspects of emotion regulation, that is, emotional experience (Gross, 2001). This allows the adjustment of emotional responses and is considered a key aspect in understanding people’s everyday emotional regulation (Gross and John, 2003). In this study, it has been defined as the use of cognitive strategies (reappraisal) deployed by teachers to manage emotional responses in the context of their teaching. It is considered an intrapersonal skill.

Empirical evidence confirms the positive role of CMTE implemented in classrooms. A study involving 189 high school teachers in Germany showed that CMTE was related to teachers’ experience of positive emotions, such as enjoyment (Lee et al., 2016); another study on 205 high school teachers also in Germany confirmed the importance of teachers’ CMTE in predicting their behaviors for effective classroom instruction (Seiz et al., 2015). However, research on how teachers regulate their emotions is still scarce, and little empirical evidence is available. This is surprising, as how teachers regulate their emotions has been recognized as a fundamental aspect in improving their effectiveness (Gross, 2002), their personal and professional success, equipping them to show empathy toward their students, highlighting its indisputable importance in school contexts (Gross, 2001; Fan and Wang, 2022).

#### 1.4.2 Teacher empathic concern

Empathy is understood as the ability to orient and respond to the thoughts, actions, feelings, and experiences of others (Coke et al., 1978; Decety and Cowell, 2014; Matravers, 2014); multidimensional in nature given that it integrates cognitive, socioemotional, and behavioral components; therefore, it can influence interpersonal and social relationships (Landler-Pardo et al., 2022; Shamay-Tsoory et al., 2009). Among the various types of empathy, empathic concern has been defined as an affective, sensitive, and compassionate response characterized by the fostering of altruistic motivation to support or help people (Batson et al., 2007; Fry and Runyan, 2018; Winczewski et al., 2016). In this study, TEC, has been defined as an emotion oriented toward students, activated by perceiving that they need something, triggering motivation, prosocial behavior, and a compassionate disposition to increase their well-being during class.

In educational settings, TEC plays an indispensable role (Landler-Pardo et al., 2022) since it is linked to the promotion of healthy intergroup relationships in the classroom (Fry and Runyan, 2018); it also allows for the deployment of empathic behaviors in complex interactions, being fundamental to consolidate strong and positive relationships with students (Landler-Pardo et al., 2022); this is why it is considered a significant component of teachers’ social–emotional learning, an important prerequisite for high-quality teacher–student interactions, development and positive outcomes of their students (Aldrup et al., 2022).

#### 1.4.3 Teacher–student relationship

Teacher–Student Relationship (TSR) are dyadic social processes characterized by continuous and feedback two-way interactions between teachers and students in classrooms that provide insight into how teachers and students feel about each other and how teachers and students perceive their shared relationships (Brinkworth et al., 2018; Cooper and Miness, 2014; Wentzel, 2022). In this study, it has been defined as an interpersonal teacher’s skill. In educational contexts, TSR is recognized as a crucial aspect of making schools inclusive and committed to providing a learning environment for healthy development and optimal learning support for all students regardless of achievement, language, readiness for learning and behavior, or disability (Pastore and Luder, 2021; Wentzel, 2022).

Empirical evidence recognizes TSR as the core of the school experience (Gehlbach et al., 2012). A longitudinal study with 1,088 German high school students showed that positive teacher–student relationships improved satisfaction of competence, relatedness, and autonomy needs (Bakadorova and Raufelder, 2018). A systematic review examining 46 studies found that high-quality teacher–student relationships are linked to higher school engagement, reflected in better academic performance, higher attendance, and decreased disruptive behaviors and dropout rates (Quin, 2017). Therefore, a strong and positive TSR is critical to emotional well-being, academic success, and engagement in learning, acting as an essential pillar for an educational environment where mutual support and understanding foster a climate conducive to learning and personal development.

#### 1.4.4 Adverse classroom climate

Classrooms are complex social systems, and the different situations generated within them are often multicomponent challenges for teachers (Pianta et al., 2012). ACC is defined in this study as the awareness of challenging situations with students in a given class according to the teacher’s self-report (behaviors, performances and/or attitudes) and corresponds to a teacher’s interpersonal skill.

Empirical evidence has shown that lower levels of conflict in the teacher–student relationship was related to higher levels of student enjoyment of learning processes (Clem et al., 2021). A study involving 267 students and 93 teachers conducted classroom climate observations revealing that emotional support favors the development of the student–teacher relationship, where students who received greater emotional support experienced closeness in the relationship and decreased conflicts with peers (Moen et al., 2019). Another study in a sample of 3,225 students from schools in Germany and Switzerland evidenced that classroom climate had a direct positive effect on counter-talk and social skills (Wachs et al., 2023). Therefore, the challenge of dealing with adversity in the classroom and the need to promote a positive climate highlight the crucial importance of teacher social–emotional development in the educational context.

### 1.5 The present study

The growing incorporation of social–emotional programs in schools underscores the need for precise methods to assess Social–Emotional Skills (SEMS) in both students and teachers. Although self-report instruments have been used for this purpose, there is a significant gap in tools specifically designed to capture the complexity of SEMS in the teaching environment, considering factors such as age, educational context, and the specific role of teachers in social–emotional development. This study set out to examine the psychometric properties of the SocioEmotional Skills Instrument for Teachers (SEMS-IT), aimed at assessing essential SEMS in teachers.

## 2 Method

An instrumental design was used to carry out the study, which, according to the classification of Ato et al. (2013), considers the analysis of the psychometric characteristics of measurement scales.

### 2.1 Sample

The total study sample consisted of 853 teachers from schools in southern Chile, with an age of M = 36.64 (SD = 10.20). Regarding sex, 610 (71.5%) were female, 229 (26.9%) were male, and 14 (1.6%) preferred not to state their sex. Two samples were used for the present study: sample 1 was composed of 468 teachers, age M = 35.79 (SD = 9.99), of whom 343 were women, 120 were men, and five indicated having another sex or preferred not to say. Regarding the declared contract hours, they had, on average, M = 35.51 (SD = 13.74) working hours per week and finally, regarding teaching experience, the participants indicated an M = 9.65 (SD = 8.74) years; and sample 2 was composed of 385 teachers of age M = 37.95 (SD = 9.79), of whom 267 indicated being female, 109 indicated being male and nine indicated having another sex or preferred not to say. Regarding reported contract hours, participants worked M = 39.89 (SD = 6.58) hours per week. Finally, participants had an average of 11.64 (SD = 8.91) years of experience.

### 2.2 Instrument

#### 2.2.1 Preliminary construction of the SocioEmotional Skills Instrument for Teachers

For the design process of this instrument, the steps for instrument construction in research (Muñiz and Fonseca-Pedrero, 2019) and the guidelines of the International Test Commission (Hernández et al., 2020) were followed. Exhaustive reviews of the specialized literature were conducted to systematize the available evidence on the history, concept, instruments, and models available on Teachers’ Social–Emotional Competence (Lozano et al., 2021, 2022, 2023; Scheirlinckx et al., 2023). From these literature reviews, dimensions and items were proposed considering specific contributions of some related instruments (Davis, 1983; Gross and John, 2003; López-Angulo et al., 2020; Pagano and Vizioli, 2021; Péloquin and Lafontaine, 2010; Zhou and Ee, 2012). As a result of this process, the first version of the SocioEmotional Skills Instrument for Teachers (SEMS-IT) was constituted as a 7-point Likert-type scale (where 1 is never and 7 is always), with 29 items distributed in four dimensions: (1) CMTE (six items); (2) TEC (eight items); (3) TSR (eight items); (4) ACC (seven items).

#### 2.2.2 Evidence of validity of the SEMS-IT

**Figure 1:**
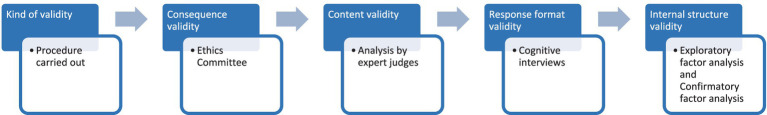
The validation process of the SocioEmotional Skills Instrument for Teachers (SEMS-IT).

The validity of an instrument is obtained when evidence and theory allow the adequate interpretation of its scores for the purpose for which it was constructed (AERA, 2014; Taherdoost, 2016); for this reason, recommendations were followed to evidence four sources of instrument validity (see Figure 1).

#### 2.2.3 Evidence of validity of consequence: ethics committee analysis

The instrument is part of a larger research project. It was submitted to the Ethics Committee of the University of affiliation of the first author of this article. The committee reviewed the instrument and the ethical protocols associated with its implementation in the target sample, to ensure the corresponding safeguards for the integrity of the participants. The respective approval was obtained.

#### 2.2.4 Evidence of content validity: analysis by expert judges

This process considered the participation of seven doctors from the areas of Education and Psychology, four from Chile and three internationals (Mexico, Ecuador, and Spain). Each expert judge evaluated the instrument using a matrix with specific criteria (pertinence, relevance, clarity, and sufficiency of each item) to measure the teaching SEMS construct and its four proposed dimensions. The inter-rater consistency results were high (Cohen’s Kappa >0.7) in the four specified criteria and all its items (Pedrosa et al., 2013; Polit et al., 2007). In addition, the wording of the items was improved when this was suggested by more than one judge.

#### 2.2.5 Evidence of response format validity: cognitive interviews

This process involved the development of cognitive interviews with nine secondary education teachers from different specialties and three schools selected by accessibility. The purpose of the cognitive interview was the identification of comprehension and/or writing difficulties of the different items (Castillo-Díaz and Padilla, 2013; Wolcott and Lobczowski, 2021). The interviews followed a protocol that considered the presentation of the study, the objective of the research project, confidentiality of personal information, instructions, clarification of doubts, and finally, the implementation of the interview. In the instruction stage, it was explained to the teachers that as they were answering the instrument, they should do so through the “thinking aloud” procedure, making observations, comments, suggestions, or consultations on the wording and/or content of the items, understanding of the instructions or the scale for answering. As a result of this process, minor changes were made to specific words in the items, such as some synonyms or adding examples in parentheses to make it easier to understand the instrument.

#### 2.2.6 Evidence of validity of factorial structure

For the third phase of structural validity of the SocioEmotional Skills Instrument for Teachers (SEMS-IT), two samples of teachers were used. The first sample (*n* = 468) was used to carry out the exploratory graph analysis (EGA). The second sample (*n* = 385) was used to conduct confirmatory factor analysis (CFA). The results section of this study presents in detail the structural validity process of this instrument.

### 2.3 Data collection procedure

The participating schools were contacted, and the principals and their respective management teams were informed about the purposes of the research and their authorization was requested. Subsequently, the schools that agreed to participate provided a contact from the management team to coordinate the presentation of the study and extend the invitation to participate to the teachers. In face-to-face meetings and/or through the Zoom platform, as decided by each school, teachers were informed of the details of the research and a deadline was coordinated for sending and responding to the instrument. The instrument was applied in online format using the surveymonkey tool. The link was sent by e-mail. The average response time was 15 min. The data were collected during the second semester of the year 2023. The questionnaire sent to the teachers had three sections: the first one displayed the informed consent; then, those who agreed to participate displayed the second part on sociodemographic data of the teachers such as for example, sex, age, employment contract (hours per week), work experience (years); and finally, the third part was the SEMS-IT instrument. All the procedures were authorized by the scientific ethical committee of the sponsoring institution of the main author of this study, guaranteeing the conditions of confidentiality, voluntariness and protection of the data obtained.

### 2.4 Data analysis procedure

A descriptive statistical analysis of the total sample was performed. Subsequently, the sample was divided into two groups, the first to examine the structure of the SEMS-IT (*n* = 468) and the second for its confirmation (*n* = 385). Statistical-descriptive analyses were performed on both samples, and the lack of differences between them was verified. First, the Kolmogorov–Smirnov test with the Lilliefors modification (samples larger than 50 subjects) was used to verify the normality assumption. Then, the homoscedasticity assumption was verified using Levene’s test. Considering that the results of this process showed that the normality assumption was not met, and neither was the homoscedasticity assumption fully met, the comparison of the two samples was performed with the Yuen test (Wilcox, 2012), being the robust alternative to the Student’s *T*-test. To estimate the effect size, we used the one proposed by Algina et al. (2005), which is the robust option to Cohen’s *d* and is interpreted in the same way. To identify the factor structure of the instrument with sample 1 (*n* = 468), we first checked the local independence assumption, which states that the variables (items) are not related after conditioning on a latent variable (redundancy), in order to avoid, for example, model misspecification and biased parameters. For this, we used the UVA Function of the EGAnet package that uses an EBICglasso.qgraph network estimation method and the weighted topological overlap (wTO) (Christensen et al., 2023). EGA was then implemented with the EGAnet library (Golino and Christensen, 2021), verifying dimensionality using a Gaussian GLASSO model and Louvain’s algorithm that determined the number of communities through a visual representation of regularized partial correlations; this procedure has demonstrated more accuracy than other exploratory methods (Christensen et al., 2020).

In the framework of network analyses, reliability was examined using two estimates (Christensen et al., 2020): (a) structural consistency, which is the proportion of times the number of dimensions derived from the initial EGA was exactly recovered in the replicated bootstrap samples and (b) item stability, which is the number of times each item is replicated within the empirical dimension and in other dimensions identified in the replication networks. Both procedures were performed with the EGAnet library and the bootEGA function (Golino and Christensen, 2021) with GLASSO estimation considering 1,000 replicates and the LE eigenvalue algorithm. For the interpretation of structural consistency and item stability, a minimum value of 90% was used; that is, the dimension was expected to replicate exactly in 90% of the bootstrap samples, and the items were expected to replicate in at least 90% of the derived dimensions (Golino and Christensen, 2021). Next, the fit of the structure suggested by EGA was verified by CFA with the CFA function of the EGAnet package (Golino and Epskamp, 2017). WLSMV estimation was used, which is suitable for scales consisting of ordinal-level items. The model was evaluated using chi-square (*χ*^2^), CFI, TLI, RMSEA, and SRMR. The criteria used to adequately evaluate the model were as follows: (a) *χ*^2^*2 p* < 0.05, (b) CFI and TLI greater than 0.9 correspond to an acceptable fit and above 0.95 to a good fit, and (c) RMSEA and SRMR with values less than 0.08 indicate an acceptable fit and less than 0.06 a good fit (Hu and Bentler, 1999). With the second sample (*n* = 385), EGA and bootEGA resampling were performed again to confirm the structural consistency and stability of the items. Finally, the results of these analyses were checked with the CFA analysis.

## 3 Results

### 3.1 Descriptive analysis of the samples

The possibility that the samples were disproportionate according to sex was analyzed with the chi-square test. The test result was *X*^2^ (3, *N* = 853) = 3.55, *p* = 0.31. Therefore, there was no evidence that the samples were unbalanced according to sex (see Table 1).

**Table 1:** Descriptive of the composition of the samples.

Sex	Sample 1	Sample 2
Man	120 (25.6%)	109 (28.3%)
Woman	343 (73.3%)	267 (69.4%)
Another	2 (0.4%)	2 (0.5%)
I prefer not to say	3 (0.6%)	7 (1.8%)
Total	468 (100%)	385 (100%)

Also, to ensure equivalence between the data of the samples, first, a descriptive analysis was performed for each item of the instrument and the variables age, contract hours, and experience. Then, to evaluate significant differences, the assumption of normality was verified, identifying that all the variables in both samples did not comply with a normal distribution (*p* < 0.05). Subsequently, the homoscedasticity assumption was verified, in this case, the variables that did not meet this assumption were: (1) EC: employment contract (hours per week), (2) TSR-17, (3) CMTE-3, and (4) CMTE-5. With this background and considering that sample 1 was larger than sample 2, the Yuen test was performed, which is a robust option of the Student’s *t*-test. For the effect size this test uses the one proposed by Algina et al. (2005), an alternative to Cohen’s *d* and interpreted on the same scale. The results showed significant differences in the variables: (1) age T (487.64) = 2.69, *p* < 0.01, ES = 0.15; (2) employment contract (hours per week) T (478.95) = 2.96, *p* < 0.01, ES = 0.16, and (3) work experience (years) T (493.24) = 3.12, *p* < 0.01, ES = 0.16. In all these cases, differences of a small magnitude were identified. On the other hand, all the items of the instrument did not show significant differences between the samples (see Table 2).

**Table 2:** Comparison of samples 1 and 2 in the study variables.

	Sample 1 (*n* = 468)					Sample 2 (*n* = 385)							
Variable	Mean	SD	Skew	Kurtosis	K-S Lilliefors	Mean	SD	Skew	Kurtosis	K-S Lilliefors	Levene	Yuen	EN
Age	35.79	9.99	0.80	0.07	D = 0.12***	37.95	9.79	0.85	0.01	D = 0.13***	F(1,851) = 0.06	T(487.64) = 2.69**	0.15
EC	35.51	13.74	−1.86	ī	D = 0.27***	39.89	6.58	−2.65	9.62	D = 0.27***	F(1,851) = 32.37***	T(478.95) = 2.96**	0.16
WE	9.65	8.74	1.23	1.22	D = 0.13***	11.64	8.91	1.18	0.95	D = 0.16***	F(1,851) = 0	T(493.24) = 3.12**	0.16
TSR9	6.32	0.81	−1.04	0.65	D = 0.31***	6.26	0.89	−1.18	1.00	D = 0.29***	F(1,851) = 0.52	T(490.84) = 0.45	
**TSR11**	**5.47**	**1.07**	**−0.52**	**0.06**	D = 0.23***	**5.50**	**1.12**	**−0.55**	**0.18**	D = 0.21***	**F(1,851) = 0.75**	**T(490.73) = 0.11**	
TSR12	6.22	1.09	−2.11	5.57	D = 0.27***	6.24	1.03	−2.10	6.33	D = 0.27***	F(1,851) = 0.06	T(490.82) = 0.09	
**TSR13**	**6.04**	**0.88**	**−0.59**	**−0.35**	D = 0.23***	**5.99**	**0.89**	**−0.59**	**−0.30**	D = 0.24***	**F(1,851) = 0.01**	**T(492.53) = 0.61**	
TSR14	6.57	0.69	−1.55	1.81	D = 0.41***	6.56	0.69	−1.56	1.94	D = 0.40***	F(1,851) = 0.06	T(489.3) = 0.29	
TSR15	5.53	1.15	−0.56	0.04	D = 0.20***	5.45	1.25	−0.72	0.09	D = 0.21***	F(1,851) = 1.08	T(420.51) = 0.30	
**TSR17**	**5.05**	**1.11**	**−0.29**	**−0.05**	D = 0.18***	**5.04**	**1.22**	**−0.49**	**0.11**	D = 0.19***	F(1,851) = 5.58*	**T(478.08) = 0.33**	
**TSR23**	**5.27**	**1.21**	**−0.57**	**0.43**	D = 0.17***	**5.30**	**1.23**	**−0.46**	**−0.25**	D = 0.20***	**F(1,851) = 2.16**	**T(480.11) = 0.16**	
**ACC10**	**2.24**	**1.31**	**1.48**	**2.21**	D = 0.29***	**2.20**	**1.32**	**1.38**	**1.91**	D = 0.23***	**F(1,851) = 1.22**	**T(473.71) = 0.19**	
**ACC16**	**2.22**	**1.11**	**1.67**	**4.15**	D = 0.30***	**2.16**	**1.00**	**1.58**	**4.47**	D = 0.29***	**F(1,851) = 0.81**	**T(489.36) = 0.05**	
**ACC18**	**2.90**	**1.26**	**0.73**	**0.68**	D = 0.19***	**2.95**	**1.26**	**0.56**	**0.22**	D = 0.18***	**F(1,851) = 0.06**	**T(487.33) = 0.85**	
**ACC19**	**3.09**	**1.37**	**0.45**	**−0.16**	D = 0.18***	**3.18**	**1.30**	**0.38**	**−0.03**	D = 0.16***	**F(1,851) = 1.86**	**T(495.75) = 1.27**	
**ACC20**	**3.59**	**1.29**	**0.35**	**−0.03**	D = 0.18***	**3.48**	**1.38**	**0.29**	**−0.31**	D = 0.15***	**F(1,851) = 3.8**	**T(418.45) = 1.17**	
**ACC21**	**2.76**	**1.40**	**1.04**	**0.82**	D = 0.25***	**2.59**	**1.33**	**1.04**	**0.72**	D = 0.27***	**F(1,851) = 1.56**	**T(498.21) = 1.75**	
**ACC22**	**2.34**	**1.30**	**1.09**	**1.04**	D = 0.26***	**2.30**	**1.17**	**0.88**	**0.50**	D = 0.26***	**F(1,851) = 1.97**	**T(495.18) = 0.48**	
**TEC1**	**6.32**	**0.85**	**−1.19**	**1.34**	D = 0.32***	**6.24**	**1.08**	**−1.78**	**3.85**	D = 0.31***	**F(1,851) = 1.35**	**T(491.61) = 0.75**	
TEC2	6.42	0.81	−1.37	1.54	D = 0.36***	6.41	0.84	−1.38	1.57	D = 0.36***	F(1,851) = 0.07	T(490.73) = 0.03	
TEC3	6.53	0.77	−1.51	1.30	D = 0.41***	6.48	0.87	−1.91	4.63	D = 0.40***	F(1,851) = 0.69	T(490.18) = 0.11	
**TEC4**	**6.53**	**0.83**	**−2.33**	**7.42**	D = 0.40***	**6.53**	**0.85**	**−2.08**	**4.59**	D = 0.40***	**F(1,851) = 0.01**	**T(492.76) = 0.40**	
**TEC5**	**6.18**	**0.94**	**−0.99**	**0.32**	D = 0.28***	**6.11**	**1.09**	**−1.42**	**2.09**	D = 0.26***	**F(1,851) = 2.25**	**T(488.42) = 0.18**	
**TEC6**	**6.52**	**0.86**	**−2.10**	**4.85**	D = 0.40***	**6.51**	**0.88**	**−1.91**	**3.18**	D = 0.40***	**F(1,851) = 0.03**	**T(491.66) = 0.18**	
TEC7	6.27	0.98	−1.37	1.45	D = 0.32***	6.21	1.00	−1.25	0.98	D = 0.29***	F(1,851) = 1.01	T(372.8) = 0.99	
TEC8	5.78	1.21	−0.83	0.08	D = 0.22***	5.60	1.29	−0.80	0.15	D = 0.22***	F(1,851) = 1.40	T(418.9) = 1.75	
**CMTE1**	**5.00**	**1.56**	**−0.85**	**0.22**	D = 0.18***	**4.83**	**1.67**	**−0.60**	**−0.35**	D = 0.16***	**F(1,851) = 2.96**	**T(484.21) = 1.94**	
**CMTE3**	**5.14**	**1.27**	**−0.44**	**−0.22**	D = 0.18***	**4.99**	**1.38**	**−0.40**	**−0.23**	D = 0.18***	F(1,851) = 4.70*	**T(478.04) = 1.83**	
**CMTE5**	**5.65**	**1.17**	**−0.96**	**1.08**	D = 0.23***	**5.56**	**1.34**	**−0.98**	**0.77**	D = 0.23***	F(1,851) = 4.59*	**T(405.36) = 0.02**	
CMTE7	5.20	1.42	−0.93	0.60	D = 0.21***	5.10	1.43	−0.73	0.15	D = 0.19***	F(1,851) = 0.01	T(487.84) = 1.39	
CMTE8	5.24	1.26	−0.73	0.46	D = 0.21***	5.25	1.25	−0.59	−0.14	D = 0.23***	F(1,851) = 0.35	T(482.71) = 0.06	
**CMTE10**	**5.34**	**1.23**	**−0.81**	**0.68**	D = 0.22***	**5.28**	**1.27**	**−0.77**	**0.38**	D = 0.23***	**F(1,851) = 0.39**	**T(483.28) = 0.57**	

SD, standard deviation; K-S Lilliefors, Kolmogorov–Smirnov with Lilliefors modification; * = *p* < 0.05; ** = *p* < 0.01; *** = *p* < 0.001; EC = employment contract (hours per week); WE: work experience (years); CMTE = cognitive management of teacher emotion; TEC = teacher empathic concern; TSR = teacher–student relationship; ACC = adverse classroom climate; In bold the items selected in the final scale after the exploratory graph analysis (EGA).

### 3.2 Results of exploratory graph analysis on sample 1

**Figure 2:**
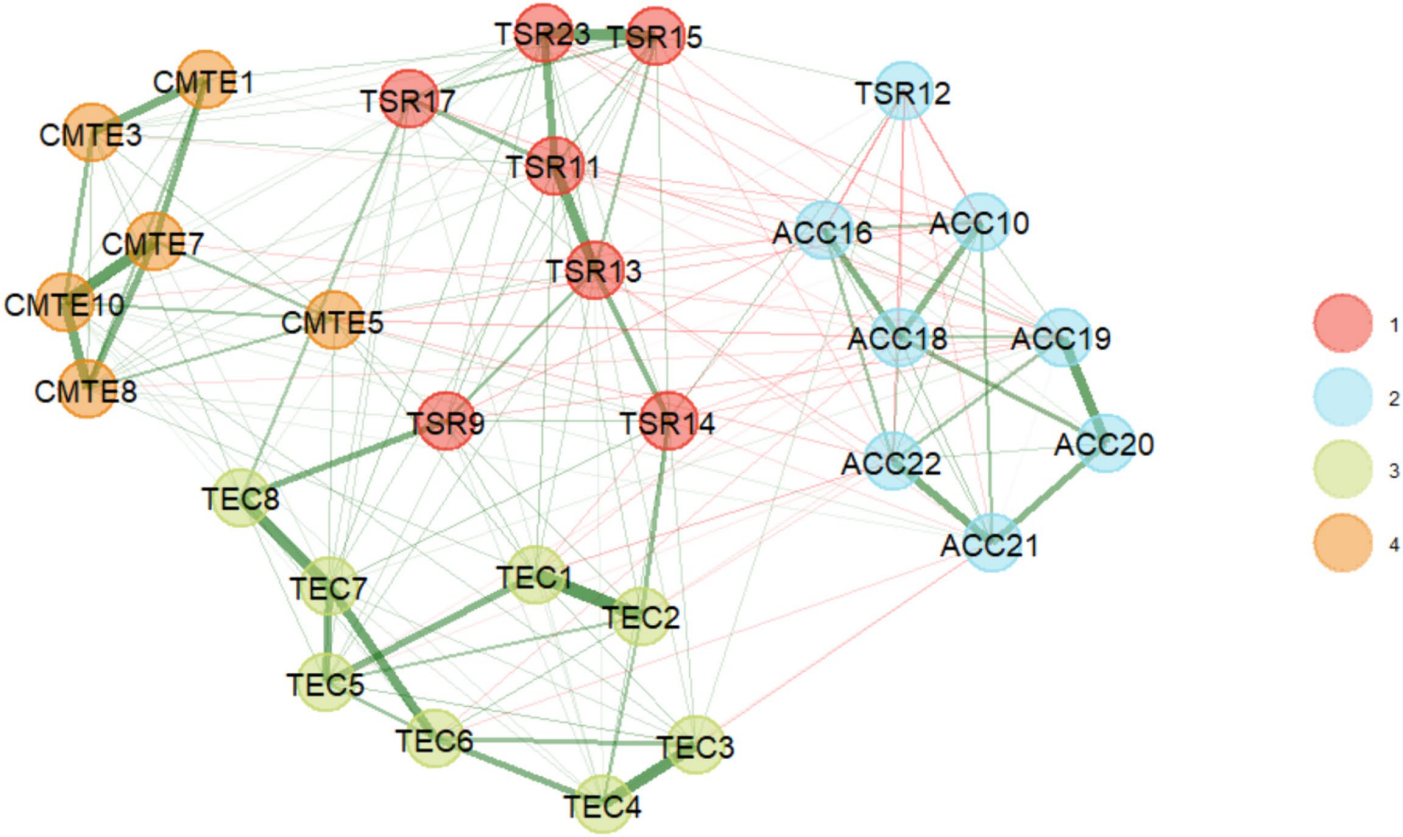
EGA dimensionality results (29 items).

Exploratory graph analysis with the GLASSO network estimation method and the Louvain community detection algorithm estimated four factors (Figure 2), representing the theoretical factors. Community 3 is consistent with the dimension “teacher empathic concern” (TEC1, TEC2, TEC3, TEC4, TEC5, TEC6, TEC7, TEC8). Community 4 is consistent with the dimension “cognitive management of teacher emotion” (CMTE1, CMTE3, CMTE5, CMTE7, CMTE8, CMTE10). Community 1 corresponds almost entirely to the dimension “teacher–student relationship” (TSR9, TSR11, TSR13, TSR14, TSR15, TSR17, TSR23), except for item TSR12, which was assigned to community 2, which corresponds theoretically to the dimension Adverse Classroom Climate (ACC10, ACC16, ACC18, ACC19, ACC20, ACC21, ACC22).

The item that presents this problem (TSR12) has the following wording “My students are uncomfortable when I give them physical affection (such as pats on the shoulder, a handshake)” one explanation may be the conflict and risks associated with the current context where physical contact could be interpreted as harassment, making it difficult for teachers to respond to this item.

The UVA analysis showed evidence of 2 pairs of items with large to very large redundancy (wTO > 0.30), four pairs of items with moderate to large redundancy (wTO > 0.25) and three pairs of items with small to moderate redundancy (wTO > 0.20). Considering this background, the strategy of eliminating redundant items was used.

**Figure 3:**
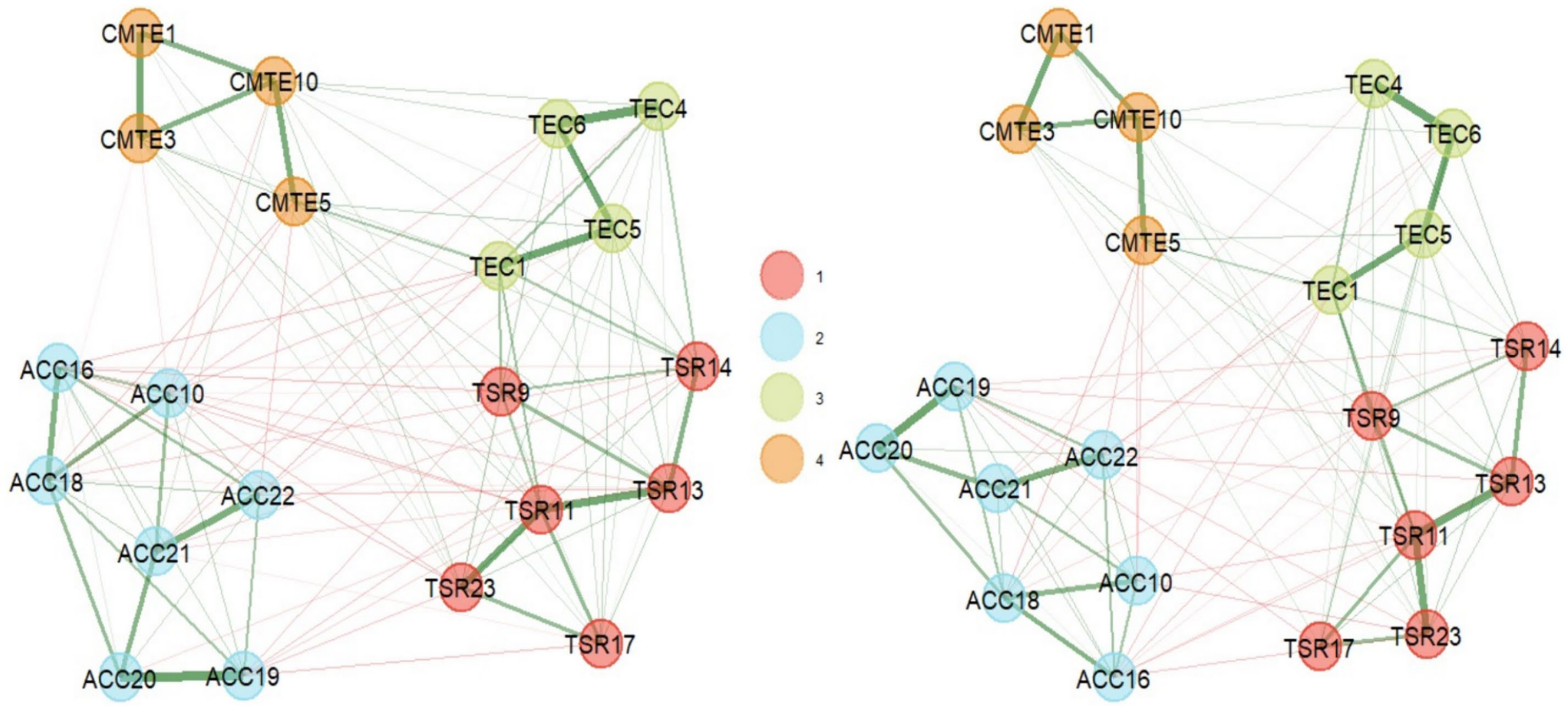
Dimensionality results in EGA (left) and bootEGA (right) 21-item questionnaire.

With this background of theoretical correspondence and local redundancy in a multivariate dataset, it is decided to eliminate the items: TEC2, TEC3, CMTE7, TEC7, TEC8, CMTE8, TSR15, and TSR12 (see Figure 3).

To evaluate the stability of these dimensions and their reproduction in a resampling, the bootEGA function with 1,000 iterations was used. Due to the ordinal nature of the Likert scales, a nonparametric resampling was used, where the network structure obtained from the median of the iterations has four dimensions, like the empirical EGA (Figure 3).

The first review of stability was performed by reviewing the descriptive statistics (Table 1). It could be observed that the median was four dimensions, the same as that reflected by the empirical EGA, together with a narrow confidence interval (95% CI [3.76, 4.24]), as a complementary measure, the frequency of each dimensional solution can be observed (Table 3).

**Table 3:** Descriptive statistics of the stability of the dimensions.

N. Boots	Median dim	SE dim	CI dim	Lower CI	Upper CI	Lower quantile	Upper quantile
1,000	4	0.12	0.24	3.76	4.24	4	4

With the frequency analysis, four dimensions were identified 98.5% of the time, which corresponds to 985 times out of 1,000 bootstrap resamples; on the other hand, three dimensions were identified 1.5% of the time, or in 15 out of 1,000 bootstrap resamples. These results suggest that the four-dimensional solution has good stability (see Table 4).

**Table 4:** The frequency of the number of dimensions in the resampling process.

No. of factors	Frequency
4	0.985
3	0.015

To obtain a better understanding of the stability of each dimension, the structural consistency, or the frequency with which the empirical EGA dimension replicated exactly in the Bootstrap resampling was calculated. The structural stability result shows that dimension 1, which represents TSR, presents a stability (0.782), being the only one lower than 0.9 (Table 5).

**Table 5:** Stability by the dimension of SEMS-IT in bootstrap resampling.

Dimension	Stability
1 (TSR)	0.782
2 (ACC)	1.000
3 (TEC)	0.977
4 (CMTE)	0.992

Then, the stability values of the items in the empirical dimensions of the EGA were observed (zero values have been eliminated to facilitate interpretability); items TSR14 and TSR9 coincided 86.5 and 81.5%, respectively, with their theoretical dimension (dimension 1), which is considered unstable, on the other hand, the items of dimension 2 representing the ACC were the only ones that in their totality presented a stability of 100% with their theoretical dimension (see Table 6). These results suggest that, although, in general, the items are associated with their theoretical dimension, there is evidence of unstable items that cause problems with the consistency of the SEMS-IT closeness dimension. With this background, we proceeded to eliminate items TSR9 and TSR14.

**Table 6:** Stability of the SEMS-IT items in bootstrap resamplings.

	1 (TSR)	2 (ACC)	3 (TEC)	4 (CMTE)
TSR11	1			
TSR13	0.986		0.014	
TSR14	0.815		0.185	
TSR17	1			
TSR23	1			
TSR9	0.865		0.135	
ACC10		1		
ACC16		1		
ACC18		1		
ACC19		1		
ACC20		1		
ACC21		1		
ACC22		1		
TEC1	0.023		0.977	
TEC4	0.015		0.985	
TEC5	0.015		0.985	
TEC6	0.015		0.985	
CMTE1				1
CMTE10				1
CMTE3				1
CMTE5		0.001	0.007	0.992

**Figure 4:**
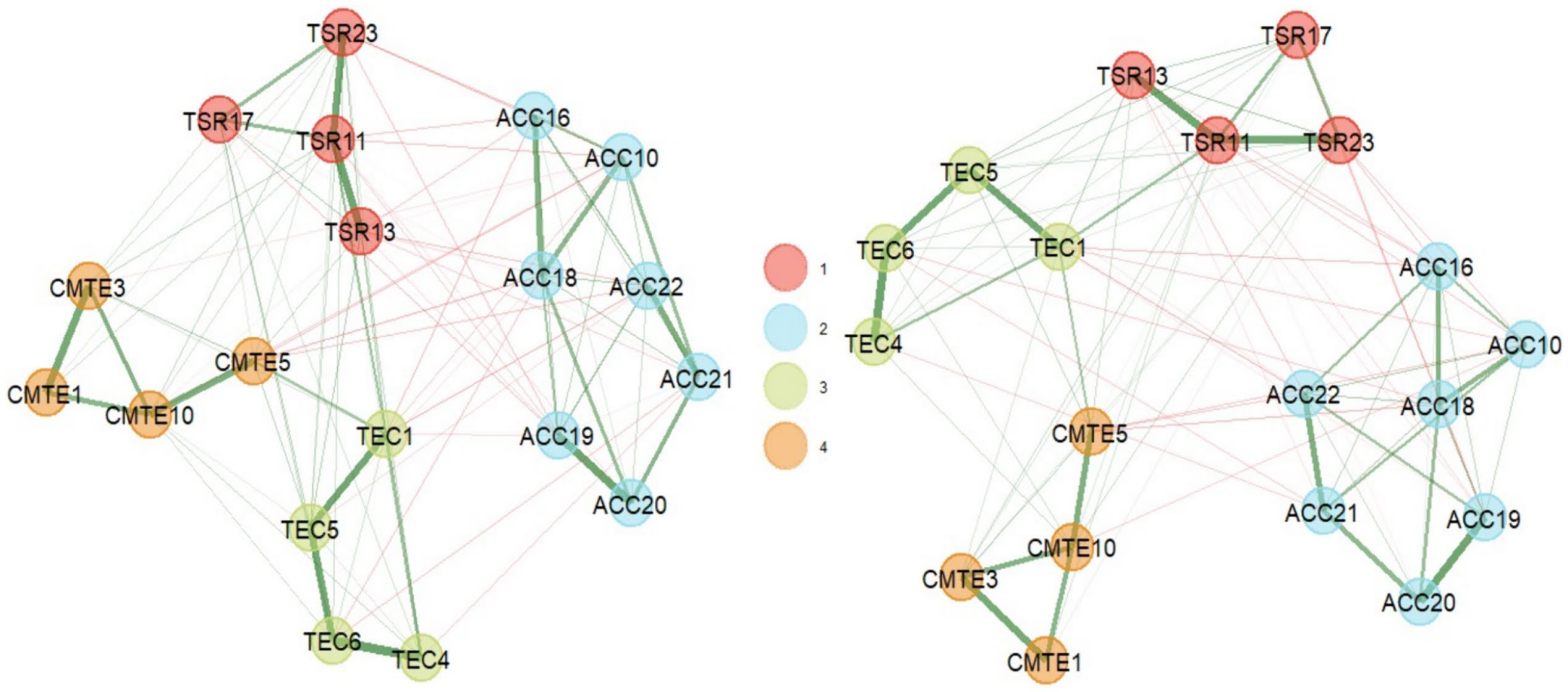
Dimensionality results of the SEMS-IT in its final solution (19 items), in EGA (left) and bootEGA (right).

Finally, in this last SEMS-IT solution of 19 items (see Figure 4), it could be observed that both the EGA analysis and the bootEGA resampling presented similar four-factor structures, where the dimension 1 corresponding to TSR was composed of the items TSR11, TSR13, TSR17 and TSR23; dimension 2 corresponding to ACC and was composed of items ACC10, ACC16, ACC18, ACC19, ACC20, ACC21, and ACC22; dimension 3 corresponding to TEC and was composed of items TEC1, TEC4, TEC5, and TEC6; finally dimension 4 corresponding to CMTE and was composed of items CMTE1, CMTE3, CMTE5, and CMTE10.

Regarding the stability of the dimensions, the frequency analysis indicated that the four-dimensional solution replicated 99.3% of 1,000 resamples, and the structural consistency or the frequency with which the empirical EGA dimension replicated exactly on resampling was 0.999 for dimensions 1 and 2, respectively, dimension 3 replicated 0.994 and dimension 4 replicated 0.988.

**Figure 5:**
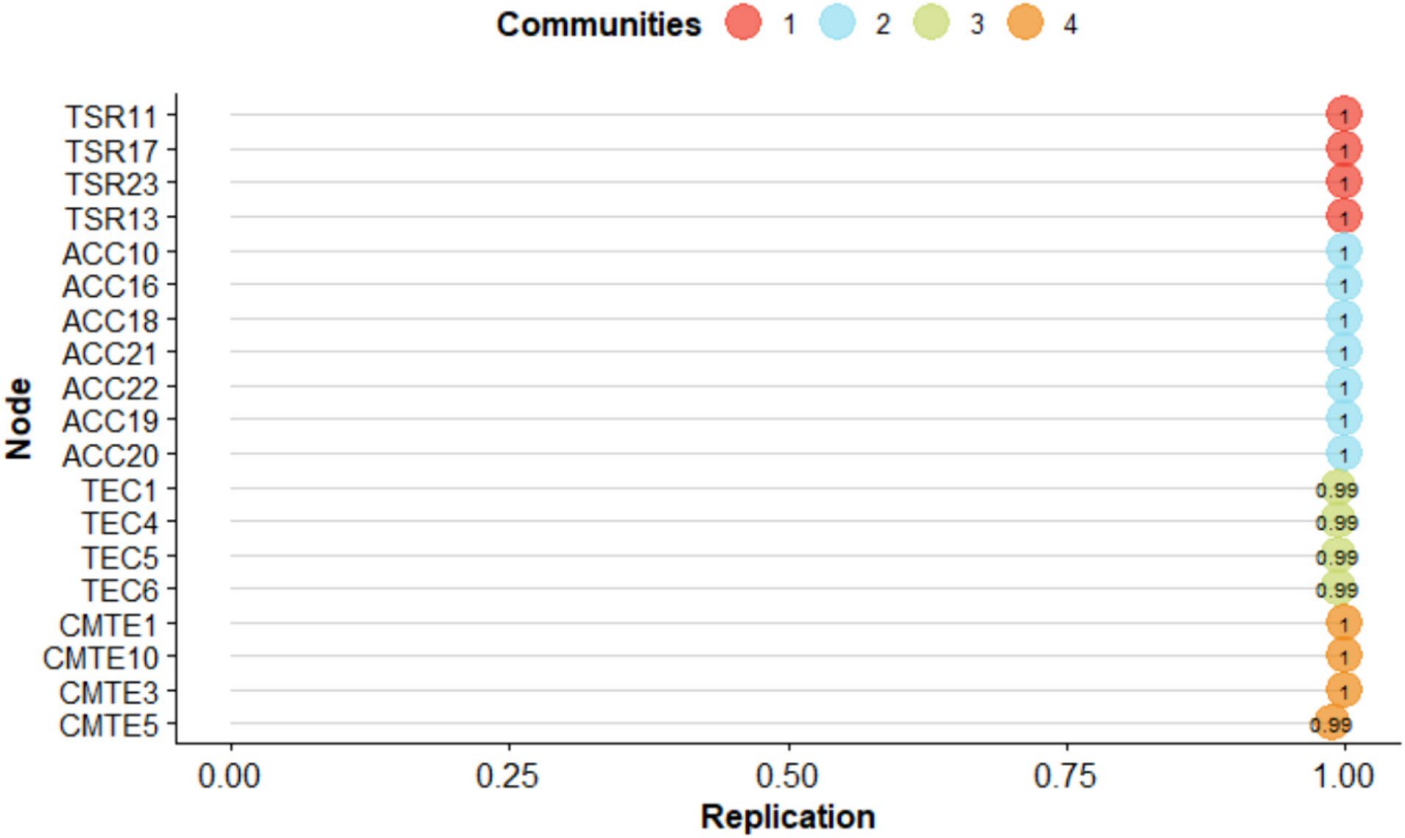
Item replication ratios for the specified dimensions of the SEMS-IT.

Then, when observing the stability of the items, it could be seen that the items were replicated in their community at least 99% of the time in their dimension (Figure 5).

**Figure 6:**
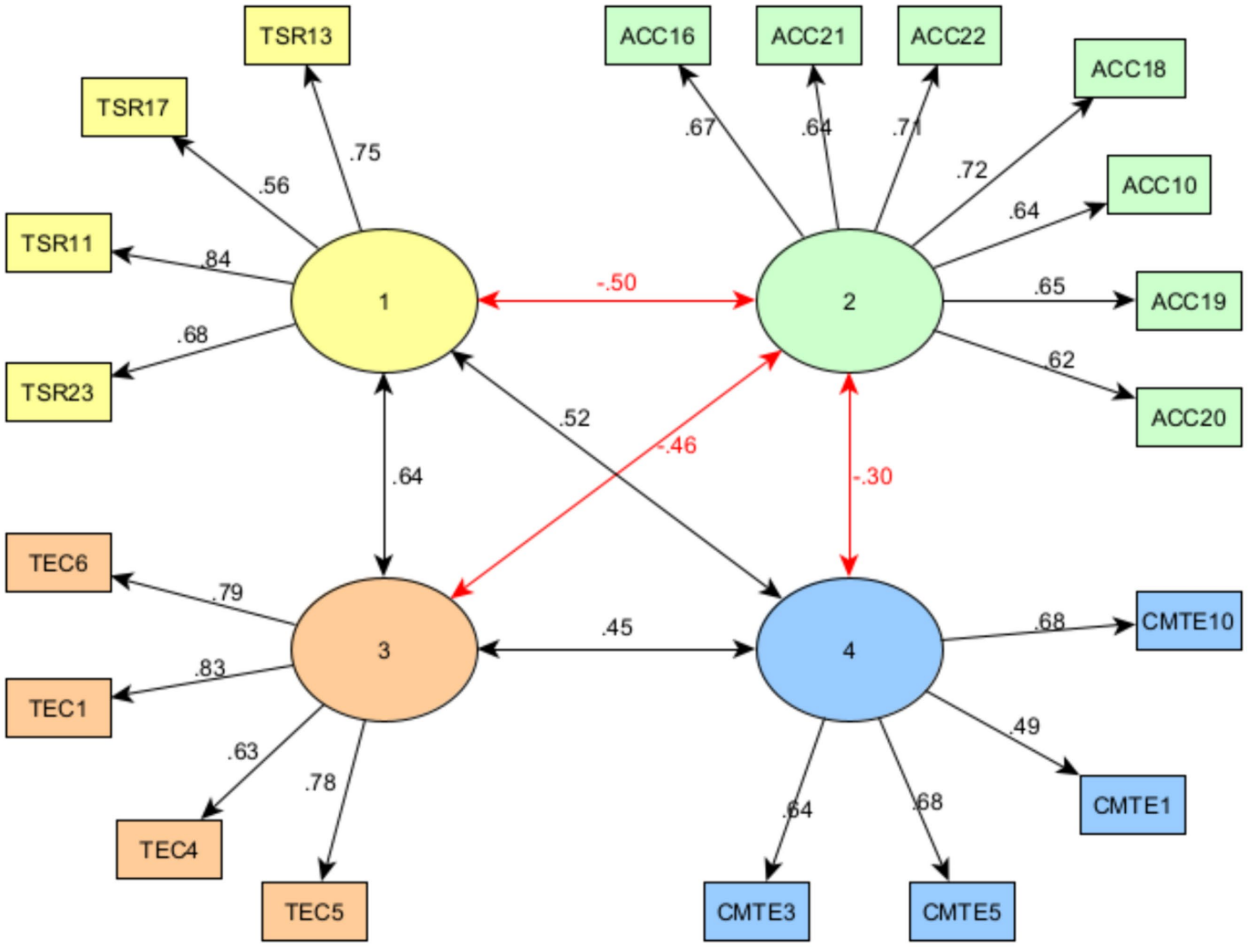
Factor structure and interaction of the SEMS-IT dimensions with latent network analysis.

Finally, the fit of the structure suggested by EGA was estimated by CFA, using the CFA function of the EGAnet package, where the model presented good fit indicators, *X*^2^ (171) = 340.926***, CFI = 0.982, TLI = 0.979, RMSEA = 0.05, and SRMR = 0.055 (Figure 6).

### 3.3 Results of the exploratory graph analysis in sample 2

**Figure 7:**
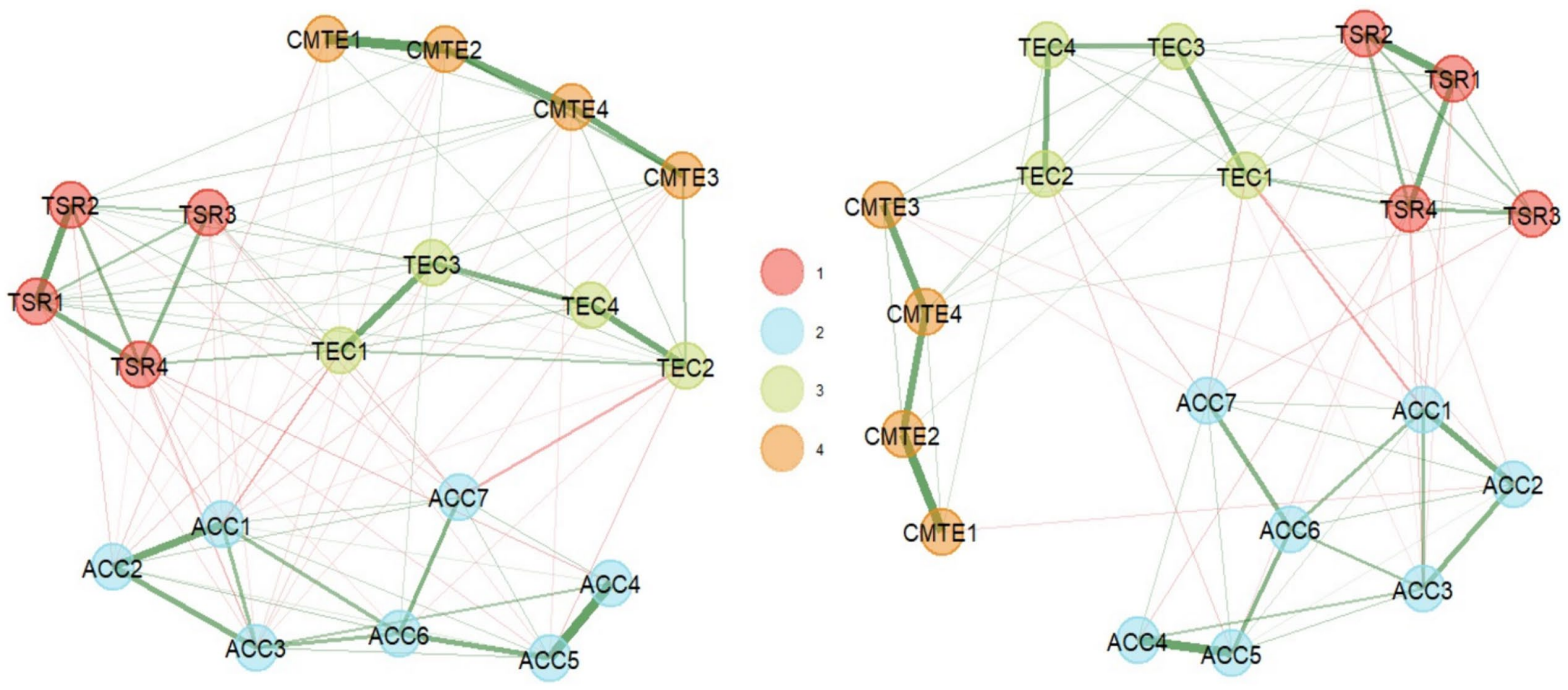
Dimensionality results in EGA (left) and bootEGA (right) of the SEMS-IT with 19 items (sample 2).

Finally, these findings were analyzed in a second sample with the items of the final solution that were selected. A new EGA was performed with the GLASSO network estimation method and the Louvain community detection algorithm, which confirmed the four-factor structure (Figure 1), consistent with the theoretical factor representation. The stability of these dimensions, as in the first part, was evaluated with the bootEGA function with 1,000 iterations, where the network structure obtained from the median of the iterations also had four dimensions, like the empirical EGA (Figure 7) has the new numbering of the items, and the adjustment of this numbering can also be seen in Appendix 1 the final scale.

**Figure 8:**
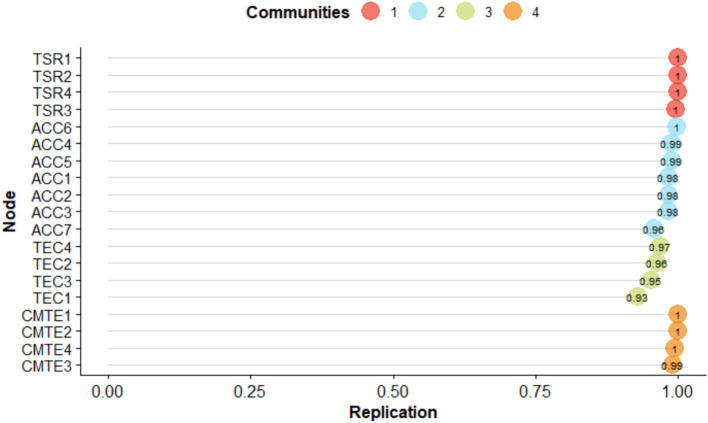
Item replication ratios for the specified dimensions.

In the frequency analysis, four dimensions were identified 94.4% of the time, corresponding to 944 times out of 1,000 bootstrap resamples. Regarding structural consistency or the frequency with which the empirical dimension of EGA replicated exactly in the resampling was 0.997 for dimension 1 (TSR), dimension 2 (ACC) replicated 0.932, dimension 3 (TEC) replicated 0.924 and dimension 4 (CMTE) replicated 0.991. Then, when analyzing the stability of the items, it could be observed that they replicated in their community at least 93% of the time (Figure 8).

Finally, the fit of the structure suggested by EGA was checked by CFA, using the CFA function of the EGAnet package. The results evidenced good and acceptable fit indicators, *X*^2^ (171) = 354.546 (*p* < 0.001), CFI = 0.971, TLI = 0.966, RMSEA = 0.061, and SRMR = 0.062.

## 4 Discussion

This research focused on examining the psychometric properties of the SocioEmotional Skills Instrument for Teachers (SEMS-IT), an instrument that includes critical and essential teacher SEMS, using the network model. This study is relevant given that SEMS are fundamental for the successful development of a person and for the effective performance of teachers. Among the most important findings, it was evidenced that the SEMS-IT presents optimal metric properties and an adequate internal structure. These results inform that the SEMS-IT can be used as a type of brief measure of SEMS in teachers.

### 4.1 Strengths of this research

One of the strengths of this research is the use of network analysis with the EGAnet package. This type of analysis provides the area of social sciences with a deeper and more integrative understanding of the structure and dynamics of the constructs being studied toward a unifying theory (Borsboom and Cramer, 2013; Lange et al., 2020). It is constituted as a psychometric network model, in this case on an instrument to measure teaching SEMS, which offers significant advantages compared to other types of analysis given that it is characterized by: (1) flexibility in representation, given that it helps to model complex relationships between different variables, which is crucial to understand the interactions between SEMS (Golino and Epskamp, 2017; Handcock et al., 2008); (2) an approach focused on network topology, which allows identifying key nodes and their connections, which is valuable for understanding how SEMS relate to each other and how patterns of influence emerge (Epskamp et al., 2017; Leskovec and Sosič, 2016); (3) the detection of spillover effects through mediating pathways in the network, for example, if one SEMS affects another through a third, the EGAnet captures this (Christensen et al., 2020); (4) robustness to missing data, since it handles them more flexibly without the need for a complete correlation matrix, and can even estimate relationships when some data are absent without affecting parameter estimation (Christodoulou et al., 2023); and (5) intuitive visualization since it provides graphical representations of the network facilitating the interpretation and communication of results. All in all, the EGAnet is considered a powerful tool in psychometric analysis (Isvoranu and Epskamp, 2023; Marsman et al., 2018; Soares et al., 2021).

Another important aspect of constructing an instrument such as this is that in the field of psychometrics, it is possible to identify measurement scales based on traits and others based on skills (Bradberry and Su, 2006; Brannick et al., 2009). However, when dealing with psychological constructs but applied in an educational performance context, it is more beneficial to have scales that facilitate the identification of categories based on people’s responses, which are possible to improve and, therefore, guide the deployment of actions for socio-emotional development. This has an important value because, even if variables with low development are identified in teachers, they have the possibility of working on them until they reach a desirable performance for the effectiveness of their classroom practice (Lee et al., 2023). The instrument has also been limited to measure teachers’ own skills in their interaction with students, highlighting its value with respect to the specificity of the constructs it measures and in a defined context, which compared to instruments that have been designed without delimiting a context, do not contribute theoretically significant amounts of variance to the models, while instruments of specific skills and specific to a context, contribute substantial amounts to the variance of the modelled predictions (Spitzberg, 1991).

### 4.2 Implications of this study at the theoretical level

This study also has strong theoretical and practical implications. First, the findings obtained may be valuable for expanding the conceptual framework of the variable SEMS teachers (Dung and Zsolnai, 2021), especially considering that the possibilities of socioemotional development depend largely on the context in which a person develops (Jones et al., 2019). The structure of individual differences in many social and emotional attributes is required to be specified, especially in teachers and their performance in the educational area, resulting in the essential to identify the main domains of socioemotional content that are required to be assessed through instruments (Primi et al., 2019). This study does not intend to propose a definitive instrument, but rather to contribute with a measurement tool that contributes to the research of teaching SEMS and encourages other instruments that complement the included domains that have been rigorously selected within the wide number of possibilities.

Specifically, we integrated as dimensions of the instrument as follows: (a) the cognitive management of emotions, as it allows teachers a cognitive change where they can modify the emotional effects before various situations in the classroom, so when they want to feel more positive emotions or less negative emotions while teaching, they can change their way of thinking about the situation (Gross, 1998; Gross and John, 2003; Hagenauer et al., 2015); (b) empathic concern, which involves teachers’ deployment of a constellation of authentic emotions when they observe needs or difficulties (personal and/or social) in their students during their teaching, allowing them to understand their thoughts and feelings by responding compassionately with sensitivity, concern, attention, sympathy, without losing sight of their students’ learning (Fry and Runyan, 2018; Bialystok and Kukar, 2018; Meyers et al., 2019); (c) the TSR, which reflects the teacher’s influence and proximity to his or her students while teaching, which has been associated with significant changes in student performance and motivation (Simon et al., 2022; Wubbels and Brekelmans, 2005); (d) adverse situations in the classroom, which allows understanding the complex and problematic situations experienced by teachers during their teaching, which are directly related to teacher burnout, symptoms of depression, stress, anxiety, low professional optimism, in turn considered critical factors in the decision of teachers to move schools or leave their profession (Agyapong et al., 2022; Burić et al., 2019; Flores, 2020; McLean et al., 2020; Papastylianou et al., 2009; Sáez-Delgado et al., 2024).

Therefore, the theoretical contribution of this study is directed toward the inquiry of those theoretical–empirical models that integrate SEMS for effective performance and high quality of teacher education (LeTendre, 2017) that allow teachers to overcome the daily difficulties in their professional practice (Fitzgerald et al., 2022; Hen and Goroshit, 2016; Jones et al., 2013), and also includes those SEMS fundamental for themselves and their students to thrive in the 21st century (Collie and Perry, 2019; Scheirlinckx et al., 2023).

### 4.3 Implications of this study at the practical level

The implications of this study at a practical level consist in the proposal of a scale that contributes to the possibility of guaranteeing greater quality and validity of the research by providing more reliable empirical evidence in the collection of data on teachers’ SEMS, especially valuable when attempting to account for the effects of interventions on SEL in teachers. This is especially relevant, given that it has been noted in the literature that studies have assessed very heterogeneous and different variables through multiple instruments that may reflect inconsistency in assessment procedures (Oliveira et al., 2021). Therefore, adequate consistency is required between the instruments selected to measure teaching SEMS with the variables to be studied, the objectives and the contents addressed in a given intervention to achieve the sensitivity of actually measuring the construct intended to be improved in the program (Domitrovich et al., 2016). In this sense, this study makes available an instrument based on the approach that has been most frequently used for the measurement of SEMS, that of self-report, because it facilitates the operationalization of the skills that are intended to be measured (Schoon, 2021). It also stands out for being a tool that adequately captures SEMS that respond to a specific context, in this case, to school-level teachers considering their formative and modelling role of these skills in their students (Jones and Bouffard, 2012; Martínez-Yarza et al., 2023; Sáez-Delgado et al., 2022).

### 4.4 Limitations and future lines of research

The results obtained provide valuable information on the validity and reliability of the SEMS-IT. However, it is important to consider some limitations and areas for future research. One aspect to consider as a limitation is related to the type of instrument used (self-report), which, especially when applied to samples of teachers, could show possible social desirability biases in the responses obtained, that is, it could be questioned whether they reach a high average in the different SEMS. There is some background on the results of teachers’ self-reports regarding their SEMS, which do not necessarily correlate with the elicitation of other data, such as physiological measures (Ciuk et al., 2015). Therefore, as a future line of research one could consider exploring other alternative or complementary measurement strategies. Within the forms/methodologies to measure socioemotional aspects, it is recognized that these vary widely, not only due to the intrinsic content of the social and emotional that is sought to be evaluated but also because some authors make a distinction between skills and competencies. Regarding the instruments for the measurement of SEMS, there are several types that have been used in empirical research highlighting the self-report, situational (Aldrup et al., 2020; MacCann and Roberts, 2008; Mayer et al., 2002) observation (Achenbach, 2019; Goodman et al., 2000; Schoon, 2021) and physiological types. Given that each of these types of instruments has strengths and limitations, future studies could consider the application of more than one of them, which would provide a deeper insight into teaching SEMS during their teaching.

Another limitation is that the participants in this study are secondary school teachers; therefore, the conclusions of this study should be applied with care to this specific group. It would be useful to replicate this study with different samples of teachers to analyze the psychometric properties of the instrument and, consequently, the generalization of the results to teachers at other educational levels. A third limitation consists of the teaching SEMS integrated into this instrument. As mentioned above, it does not seek to be a unique instrument, but rather, a relevant measurement resource to measure some key and necessary SEMS to analyze in teachers; undoubtedly, future studies could explore other SEMS that could be included, in the extent that those relevant to teachers and their socioemotional challenges in the classroom are identified. A fourth limitation is important to mention, and this one refers to the lack of a specific analysis based on the network approach that allows analyzing the relationship between the different dimensions that make up the instrument with other related variables; however, this also allows guiding a path for future research, that is, exploring the relationships between these socioemotional skills with other constructs of teachers, and also of students (Palikara et al., 2021). This will strengthen the evidence and understanding of the role that these dimensions play in the outcomes of greatest interest regarding teacher effectiveness (Bardach et al., 2022).

Finally, researchers are encouraged to use this instrument not only for the diagnosis or description of teachers’ SEMS but also in intervention studies that seek to improve SEMS, considering that educators’ own social and emotional skills play an important role in the quality of the educational experiences they offer to their students. The issue of measuring SEMS has become an increasingly urgent concern among both researchers and school leaders, who are more frequently beginning to integrate social–emotional work into classrooms to experiment with and replicate the promising effects reported in research. This is where the present study becomes very valuable to be able to evaluate the effect of interventions or teacher socioemotional development, using a sensitive measurement instrument capable of accurate assessment, which will allow causal inferences to be made about change over time in teacher socioemotional indicators (LeTendre, 2017).

In conclusion, this study contributes to the existing literature on teacher SEMS and provides a useful tool for its evaluation. The findings may have significant implications for teacher training and professional development, as well as for promoting positive and effective learning environments.

## Data Availability

The raw data supporting the conclusions of this article will be made available by the authors, without undue reservation.
