# Supplementary material for: Psychometric properties of the SocioEmotional Skills Instrument for Teachers using network approach: English and Spanish version
Source: Front Psychol. 2024 Sep 20;15:1421164. doi: 10.3389/fpsyg.2024.1421164 (PMC11449755; doi:10.3389/fpsyg.2024.1421164)
Supplement: Supplementary file 1 [file Data_Sheet_1.pdf]

Appendix 1. Final structure of the SocioEmotional Skills Instrument for Teachers (SEMS-IT)

Response format [Formato de respuesta]

To answer consider a scale from 1 to 7 where 1 is never and 7 is always [Para responder considere una escala de 1 a 7 donde 1 es nunca y 7 es siempre]:

1	2	3	4	5	6	7
Never [nunca]	Almost never [casi nunca]	Seldom [pocas veces]	Half the time [la mitad de las veces]	Frequently [con frecuencia]	Almost always [casi siempre]	always [siempre]

**Cognitive Management of Teacher Emotion - CMTE [Gestión cognitiva de la emoción del docente]**									
Original Identifier	New Item Identifier	Answer how often in your classroom interaction with your students you make the following statements *[Responda con qué frecuencia en la interacción con sus estudiantes en el aula realiza las siguientes afirmaciones]*							
CMTE1	CMTE1	When I want to feel more positive emotions in the classroom (such as joy or enthusiasm), I change what I am thinking *[Cuando quiero sentir más emociones positivas en el aula (como alegría o entusiasmo), cambio lo que estoy pensando]*	1	2	3	4	5	6	7
CMTE3	CMTE2	When I want to feel less negative emotions in the classroom (such as anger or sadness), I change what I am thinking *[Cuando quiero sentir menos emociones negativas en el aula (como enojo o tristeza), cambio lo que estoy pensando].*	1	2	3	4	5	6	7
CMTE5	CMTE3	When I find myself in a stressful situation in the classroom, I try to think about it in a way that allows me to be calm *[Cuando me encuentro en una situación estresante en el aula, trato de pensar en ella de un modo que me permita estar en calma].*	1	2	3	4	5	6	7
CMTE10	CMTE4	When I want to feel a less negative emotion in the classroom, I change the way I am thinking about the situation *[Cuando quiero sentir una emoción menos negativa en el aula, cambio la manera en la que estoy pensando sobre la situación].*	1	2	3	4	5	6	7
**Teacher Empathic concern - TEC [**Preocupación empática docente]									
Original Identifier	New Item Identifier	Please answer how often in the classroom you make the following statements. *[Responda con qué frecuencia en el aula realiza las siguientes afirmaciones]*							
TEC1	TEC1	I often worry about my students who are in a vulnerable situation (emotional, social, economic) *[A menudo me preocupo de mis estudiantes que están en una situación vulnerable (emocional, social, económica)]*	1	2	3	4	5	6	7
TEC4	TEC2	I intercede when I see a student undermining himself or herself during class *[Intervengo cuando veo que un estudiante se menoscaba durante la clase].*	1	2	3	4	5	6	7
TEC5	TEC3	My students' problems often concern me *[Los problemas de mis estudiantes suelen preocuparme].*	1	2	3	4	5	6	7
TEC6	TEC4	When I see a student being treated unfairly, I am disturbed by the situation *[Cuando veo que un estudiante está siendo tratado injustamente, me conmueve la situación]*	1	2	3	4	5	6	7
	**Teacher-student relationship -TSR [Relación docente-estudiante]**								
Original Identifier	New Item Identifier	*Answer the frequency of the following statements. In interactions I notice when: [Responda la frecuencia de las siguientes afirmaciones. En las interacciones me doy cuenta cuando:]*							
TSR11	TSR1	My students seek my help when they feel upset in the classroom *[Mis estudiantes buscan mi ayuda si se sienten disgustados en el aula]*	1	2	3	4	5	6	7
TSR13	TSR2	My students rate their relationship with me positively in the classroom *[Mis estudiantes valoran positivamente su relación conmigo en el aula]*	1	2	3	4	5	6	7
TSR17	TSR3	My students spontaneously share information about themselves during class *[Mis estudiantes comparten espontáneamente información sobre ellos mismos durante la clase].*	1	2	3	4	5	6	7
TSR23	TSR4	My students openly share their feelings and experiences with me during classes *[Mis estudiantes comparten abiertamente sus sentimientos y experiencias conmigo durante las clases]*	1	2	3	4	5	6	7
**Adverse Classroom Climate – ACC [Clima de Aula adverso]**									
Original Identifier	New Item Identifier	*Answer the frequency of the following statements. In interactions I notice when: [Responda la frecuencia de las siguientes afirmaciones. En las interacciones me doy cuenta cuando:]*							

ACC10	ACC1	*My students and I seem to be at odds when we interact with each other [Mis estudiantes y yo parece que estuviéramos enfrentados cuando interactuamos]*	1	2	3	4	5	6	7
ACC16	ACC2	My students get angry with me during class *[Mis estudiantes se enfadan conmigo durante la clase]*	1	2	3	4	5	6	7
ACC18	ACC3	My students remain angry or defiant after I have admonished them *[Mis estudiantes permanecen enfadados o desafiantes después de haberlos amonestado]*	1	2	3	4	5	6	7
ACC19	ACC4	I find it exhausting interacting with my students in the classroom *[La interacción con mis estudiantes en el aula me resulta agotadora]*	1	2	3	4	5	6	7
ACC20	ACC5	My students are in a bad mood, and I know we are going to have a long and difficult class] *[Mis estudiantes están de mal humor y sé que vamos a tener una clase larga y difícil]*	1	2	3	4	5	6	7
ACC21	ACC6	My students' feelings toward me are unpredictable or can change suddenly during class *[Los sentimientos de mis estudiantes hacia mí son impredecibles o pueden cambiar de repente durante la clase]*	1	2	3	4	5	6	7
ACC22	ACC7	My students are manipulative with me during class *[Mis estudiantes son manipuladores conmigo durante la clase]*	1	2	3	4	5	6	7

Note: Items in Spanish are in brackets.
